# Profiling single-guide RNA specificity reveals a mismatch sensitive core sequence

**DOI:** 10.1038/srep40638

**Published:** 2017-01-18

**Authors:** Ting Zheng, Yingzi Hou, Pingjing Zhang, Zhenxi Zhang, Ying Xu, Letian Zhang, Leilei Niu, Yi Yang, Da Liang, Fan Yi, Wei Peng, Wenjian Feng, Ying Yang, Jianxin Chen, York Yuanyuan Zhu, Li-He Zhang, Quan Du

**Affiliations:** 1 State Key Laboratory of Natural and Biomimetic Drugs, School of Pharmaceutical Sciences, Peking University, Beijing 100191, China; 2 Biomics Biotechnologies co. Ltd, Nantong 226016, Jiangsu Province, China; 3 Small RNA Technology and Application Institute, Nantong University, Nantong 226016, Jiangsu Province, China; 4 Department of Stomatology, Beijing Friendship Hospital, Capital Medical University, Beijing 100050, China

## Abstract

Targeting specificity is an essential issue in the development of CRISPR-Cas technology. Using a luciferase activation assay, off-target cleavage activity of sgRNA was systematically investigated on single nucleotide-mismatched targets. In addition to confirming that PAM-proximal mismatches are less tolerated than PAM-distal mismatches, our study further identified a “core” sequence that is highly sensitive to target-mismatch. This sequence is of 4-nucleotide long, located at +4 to +7 position upstream of PAM, and positioned in a steric restriction region when assembled into Cas9 endonuclease. Our study also found that, single or multiple target mismatches at this region abolished off-target cleavage mediated by active sgRNAs, thus proposing a principle for gene-specific sgRNA design. Characterization of a mismatch sensitive “core” sequence not only enhances our understanding of how this elegant system functions, but also facilitates our efforts to improve targeting specificity of a sgRNA.

With the development of CRISPR-Cas9 system, researchers are able to induce specific double-stranded breaks in chromosomal DNA, whose repair leads to either local mutations or homologous recombination with donor DNA. In most CRISPR-Cas systems, Cas9 endonuclease from *Streptococcus pyogenes* is used, which prefers a protospacer adjacent motif (PAM) of NGG and a guide RNA of 20 nucleotides^1^,^2^,^3^. In the process of the RNA-mediated DNA targeting, endonuclease Cas9 is guided to a specific target DNA by means of sequence complementarity between a single-stranded guide RNA (sgRNA) and target DNA. Under certain scenarios, sgRNA may guide Cas9 to recognize and cleave imperfectly matched DNAs, this leads to so called “off-target” cleavage. As a major concern in the development of CRISPR-Cas9 technology, off-target activity can cause genome instability and disruption of normal gene functions. Initial studies revealed that imperfectly matched DNAs with up to 6 mismatches were efficiently cleaved, causing evident off-targeting effects^4^,^5^. Using a battery of endogenous loci-targeting sgRNA, recent studies reported that while some mismatched genome loci were cleaved as efficiently as that of perfectly matched target^6^,^7^, many single-nucleotide mismatched targets however resist cleavage by an active sgRNA^8^,^9^. Besides revealing widespread off-target effects, these studies also demonstrated that some mismatches are not tolerated by CRISPR-Cas system. Furthermore, conformation of the HNH nuclease domain has been suggested to contribute to DNA cleavage activity^10^.

More recently, several groups reported to solve the crystal structure of *Streptococcus pyogenes* Cas9, complexed with guide sgRNA and target DNA^11^,^12^,^13^. In which, a bilobed architecture was revealed for Cas9 endonuclease, composing of a target recognition lobe and a nuclease lobe. The recognition lobe is essential to sgRNA and DNA binding, the nuclease lobe contains two nuclease domains and a PAM-interacting domain^11^. In the initiation of RNA-guided DNA targeting, Cas9 interacts with the PAM-proximal region and the repeat: anti-repeat duplex of sgRNA, assembling a Cas9-sgRNA binary complex. The binary complex subsequently interacts with a target DNA with a sequence complementary to a 20-nt guide region of sgRNA, to form a Cas9-sgRNA-target DNA ternary complex. Upon the assembly of the ternary complex, the two nuclease domains approach target DNA and mediate a specific double-stranded cleavage^13^.

Despite these insights gained in the functional and structural analyses, a comprehensive specificity profile is still missing. Taking advantage of a sensitive luciferase activation assay, targeting specificity of CRISPR-Cas9 system was systematically investigated in the present study.

## Results and Discussion

For genome engineering purposes, utility of CRISPR-Cas system relies on accurate selection of target DNA, in which process three components are engaged, including Cas9 protein, sgRNA and target DNA. Correct target selection derives from the base-pairing between a 20-nt sgRNA sequence and the DNA, as well as the presence of a PAM sequence. To mimic the situation in off-target cleavage, researches mutate either the sgRNA or the target DNA, to generate mismatches in the base-pairing of sgRNA and target DNA. Considering the facts that sgRNA is assembled into Cas9 endonuclease prior to its interaction with target DNA, as well as the assembly of sgRNA causes Cas9 to take a dramatic conformational rearrangement^12^, we prefer to mutate target DNA instead of sgRNA in the present study. Compared to mutating sgRNA, mutating target DNA will not affect the Cas9 assembly process of sgRNA. This, on the one hand, provides a better simulation for *in vivo* situation; and on the other hand, enables us to focus on the effects of target mismatch.

**Figure 1:**
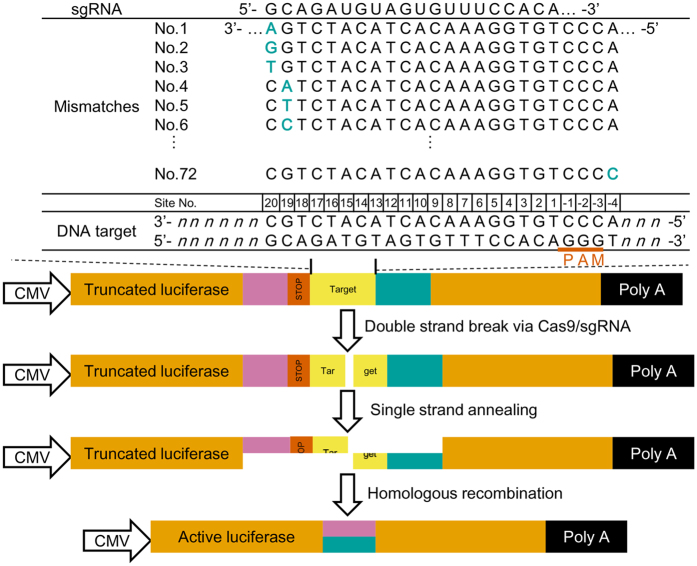
Schematic of the luciferase activation assay. sgRNA target sites carrying all possible single nucleotide mutations were tested for potential off-target effects.

To precisely evaluate the effects of target mutation in off-target cleavage, a single-strand annealing recombination assay and a luciferase reporter have been used (Biomics Inc, China). Previous research has used this robust process to investigate the activity of zinc finger nucleases (ZFNs) and transcription activated-like effector nucleases (TALENs)^14^. The system consists of three plasmids, a pCas9 plasmid encoding a cas9 endonuclease, a psgRNA plasmid encoding sgRNA sequence, and a pTarget plasmid encoding an inactive form of *firefly* luciferase reporter gene. In its inactive form, the luciferase reporter is divided into two segments, separated by an in-frame stop codon and a sgRNA target site. To enable a recombination after Cas9 cleavage, both of the segments contain a 1000 bp homologous region in direct repeat orientation. When sgRNA/Cas9 complex mediates a double-strand cleavage between the two segments, the homologous regions will initiate an efficient homologous recombination, and lead to the formation of an active *firefly* luciferase reporter (Fig. 1). Using inactive luciferase as reference, activation of luciferase gene can be quantitatively measured by assessing the gain of luciferase signals in sgRNA-treated cells. This sensitive luciferase activation assay enables us to detect subtle changes of the cleavage activity.

To assess sgRNA specificity in human cells, cleavage specificity of six effective sgRNAs were tested with all possible single-nucleotide mutated target sites. Mutations were systematically made at every position across the 20-nt target site, as well as the PAM region and 1-nt downstream PAM (Fig. 1). For each sgRNA, 73 synthetic DNA fragments bearing original or mutated target site were synthesized and cloned into pTarget vector (Biomics Inc), generating a set of target plasmid carrying all single-nucleotide mutation. The resulting plasmids were then transfected into human embryonic kidney 293 A cells (HEK293), together with pCas9 plasmid, psgRNA plasmid and a reference *Renilla* luciferase plasmid. By means of dual-luciferase assay, the activity of *firefly* luciferase was quantified, and was used to evaluate the target cleavage efficacy of a sgRNA.

**Figure 2:**
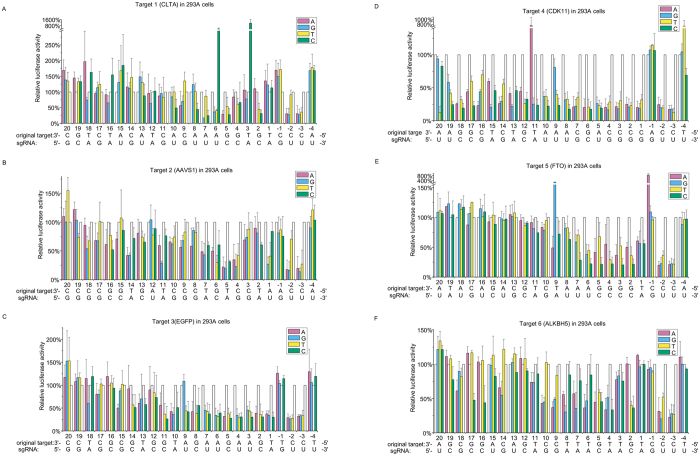
The mutation discrimination profile across sgRNA target site. Relative luciferase activities of sgRNA were plotted in terms of position and then the identity of the mutated nucleotide in sgRNA target sites. Target positions were numbered in the 5′ to 3′ direction of the DNA sequence, starting from the nucleotide immediately downstream of PAM complementary sequence. The positions of PAM region were accordingly numbered as −1, −2 and −3. Horizontal axis, sgRNA target site; vertical axis, relative luciferase activity displayed in percentage. White columns, normalized luciferase activity of original target. Scrambled target site was used as negative control. (**A**) Relative luciferase activities of sgRNA-1. Relative luciferase activity of negative control is 10.9%. (**B**) Relative luciferase activities of sgRNA-2. Relative luciferase activity of negative control is 22.4%. (**C**) Relative luciferase activities of sgRNA-3. Relative luciferase activity of negative control is 21.9%. (**D**) Relative luciferase activities of sgRNA-4. Relative luciferase activity of negative control is 10.4%. (**E**) Relative luciferase activities of sgRNA-5. Relative luciferase activity of negative control is 8.9%. (**F**) Relative luciferase activities of sgRNA-6. Relative luciferase activity of negative control is 14.6%. The data were average values of three biological replicates and every assay was conducted in triplicates.

Initial experiments revealed that, all the six sgRNAs were able to mediate efficient cleavage on their perfectly matched targets, leading to an increase of luciferase signal up to 10-fold. For the sake of simplicity, cleavage of the perfectly matched target was used as a reference and set as 100% in the study. Relative to the perfectly matched reference target, background luciferase activities were measured with scrambled target sites. For sgRNA 1–6, this led to background luciferase activity of 10.9%, 22.4%, 21.9%, 10.4%, 8.9% and 14.6%. With a reference of matched targets, relative luciferase activity of mutated targets was normalized and aligned in terms of the position and the identity of the mismatched target nucleotide (Fig. 2).

PAM recognition is a critical step in DNA targeting and cleavage^12^. In type II CRISPR system, Cas9 endonuclease was shown to bind to target site by recognizing a dominant PAM sequence of NGG^15^. In our study, analysis of PAM showed that while mutations at position −1 were generally tolerated, mutations at position −3 were not tolerated at all, leading to greatly compromised cleavage efficacies. For sgRNA-1, sgRNA-2, sgRNA-5 and sgRNA-6, when nucleotide C was mutated to T at position −2 in complementary strand and generated a PAM sequence of NAG in non-complementary strand, the cleavage efficacy was decreased relative to that of the NGG PAM, they are however much higher than that of the NTG or NCG PAM. This demonstrated that, at least for some sgRNAs, both NGG and NAG PAM sequence can be recognized and cleaved by Cas9. While for sgRNA-3 and sgRNA-4, NAG PAM could not be recognized. Together with published study^16^, these findings on one hand raise more concerns over off-target effects in CRISPR/Cas9-mediated genome engineering, on the other hand greatly expand potential targets for genome editing, especially when Cas9 is modified using structural information and improve its specificity^17^.

In consistence with previous reports^7^,^9^, our study also showed that the entire 20-base-pair target site can contribute to target specificity to some extent, depending on the sequence of sgRNA. While the cleavage efficacy varied with the position and the identity of the mismatched target nucleotide, positional effects were the most profound. Along the length of sgRNA, while modest changes in cleavage efficacy were revealed for the PAM-distal mismatches, profound effects were shown for some of the PAM-proximal mismatches. These results indicated that PAM-proximal mismatches are less tolerated than PAM-distal mismatches, and confirmed the existence of a “seed” region as previously proposed^18^,^19^.

Actually, the seed sequence of the CRISPR-Cas system has been known for quite some time^20^. Cas9 is guided by sgRNAs containing an uninterrupted 12 nt seed sequence at the 3′ end of the spacer segment^5^. Recently, structural analyses revealed that when complexed with Cas9, the entire 20-nt guide RNA segment is engaged in an A-form helical interaction with a target DNA strand^11^. Proper orientation of this structure in Cas9 endonuclease will correctly positions target DNA for cleavage by the nuclease domains. Therefore, maintaining an intact A-form conformation is critical for target recognition and cleavage. This might explain the fact that the entire 20-base-pair target site can contribute to target specificity, although with varying significance.

**Figure 3:**
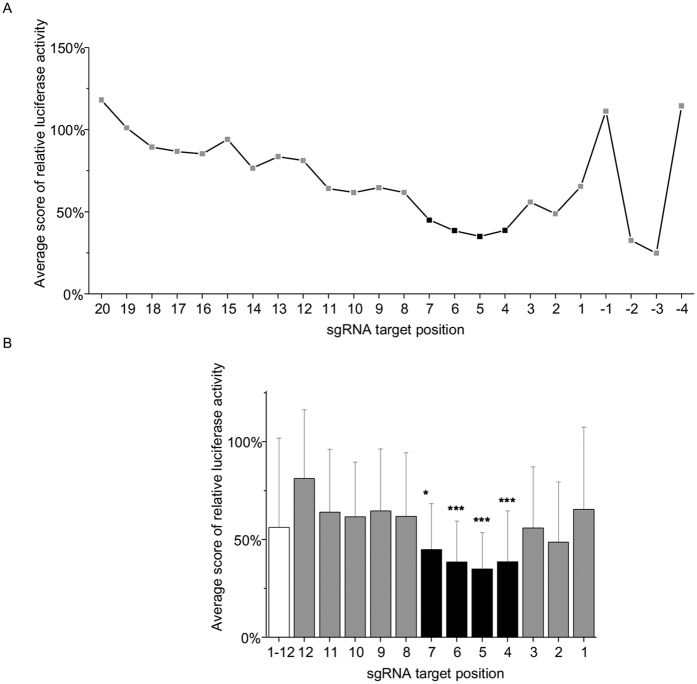
The tolerance profile of sgRNA target mutation. (**A**) A tolerance profile is obtained by averaging the relative luciferase activity of all sgRNA target sequences. (**B**) Target specificity of ‘seed sequence’. A comprehensive tolerance of target mutation is calculated by averaging the relative luciferase activity of all target positions. In relative to this reference, target specificity of seed sequence is obtained by means of Mann-Whitney test. *p < 0.05; ***p < 0.001.

In our study, while the cleavage efficacy varied with the position and the nucleotide identity in the seed region depending on the sgRNA sequence, the most profound compromising effects were surprisingly observed within a short “core” sequence, ranging from positions +4 to +7 (Fig. 3). Target cleavage was abolished by most of the single-nucleotide mismatches at this “core” sequence, rendering Cas9 activity highly sensitive to the mismatches. This also suggests a strict requirement of an intact A-form architecture for this region, which might attribute to its spatial restriction within Cas9. In structural analyses^13^, the seed region was shown to thread through a narrow nucleic acid-binding channel formed between the two Cas9 lobes. While the terminal nucleotides +1, +2, and + 8 to +10 are exposed to bulk solvent, the internal nucleotides + 3 to +7 are shielded from solvent by helical protein domains. Being located in such a spatial restricted region may render the “core” sequence subject to steric hindrance. This may further limit its conformation flexibility, and lead to a high sensitivity to target mismatch. Taken together with published studies^11^, our data characterized a mismatch-sensitive “core” sequence in sgRNA, which might be a critical determinant of targeting specificity.

In contrast to the unpredictable chromatin accessibility and the limited endogenous editing efficiency of CRISPR/Cas9 system, reporter gene assays used in the study are very sensitive and reproducible, enabling us to detect the minor functional difference of different target positions.

**Figure 4:**
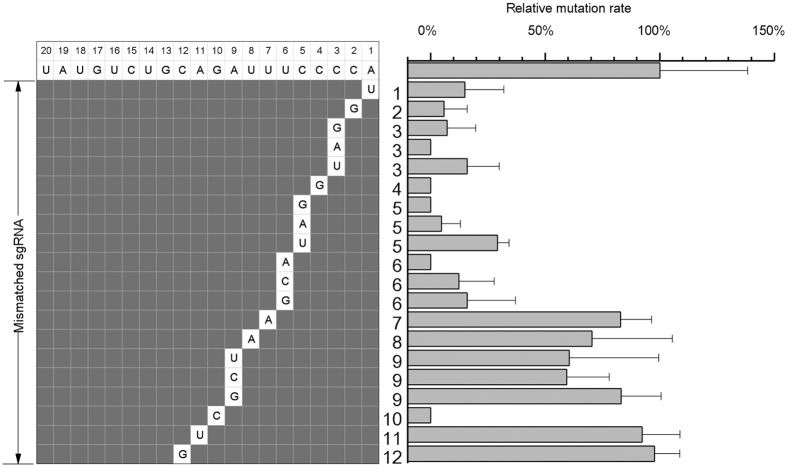
Activity of mismatched sgRNA-5 on endogenous target. Relative mutation rate of target DNA was measured by T7E1 assay. The identity and the position of the mismatched nucleotide are indicated.

To confirm the sensitivity of the core sequence, assays were conducted to target endogenous FTO. Within the seed region of sgRNA-5, position +5, +6 (core sequence) and +3, +9 (seed region but not core sequence) were randomly chosen to generate all possible single nucleotide mismatches, while single mismatch was generated for the other position. In agreement with our previous findings, T7E1 assays showed that mismatches at position +4, +5 and +6 almost abolished the cleavage activity of CRISP-cas9 system (Fig. 4).

Besides the positional effects, differential cleavage efficacies were revealed for different target mismatches, even at the same position. As for position +7 of sgRNA-1, while rU:dT mismatch was tolerated, rU:dC and rU:dG mismatches exhibited greatly compromised cleavage efficacies (Fig. 2A).

Considering a strong correlation between genome editing efficiency and sgRNA GC content of the six PAM-proximal nucleotides^21^,^22^, we examined if sgRNA specificity is also related to the GC content of target sequence. In contrast to sgRNA-1, sgRNA-2, sgRNA-5 and sgRNA-6, almost all of the seed mismatches were untolerated for sgRNA-3 and sgRNA-4, abolishing the cleavage activity on mismatched target sites. That is to say, specificity of sgRNA-3 and sgRNA-4 are much higher than that of others. As to GC content of the PAM-proximal 6-nt, no obvious correlation was revealed.

To our surprising, assays of some target mutations demonstrated significantly enhanced cleavage activity. When the matched nucleotides at target position +3 and position +6 of sgRNA-1 were mutated to cytosine, cleavage efficacies were increased by a 11.4 and 3.6-fold, respectively. Even though both of the mismatched targets have cytosine nucleotide at the mismatched position, this effect was however not observed at the same positions of other sgRNAs. Meanwhile, rA:dA at site No. 11 of target 4 and rA:dG at site No. 9 of target 5 also reveal enhanced cleavage activity. Besides our observation, this phenomenon was also reported by other groups^7^,^8^. However, further investigation on this issue revealed no obvious rule or potential mechanisms, indicating that additional efforts are needed to clarify this observation.

**Figure 5:**
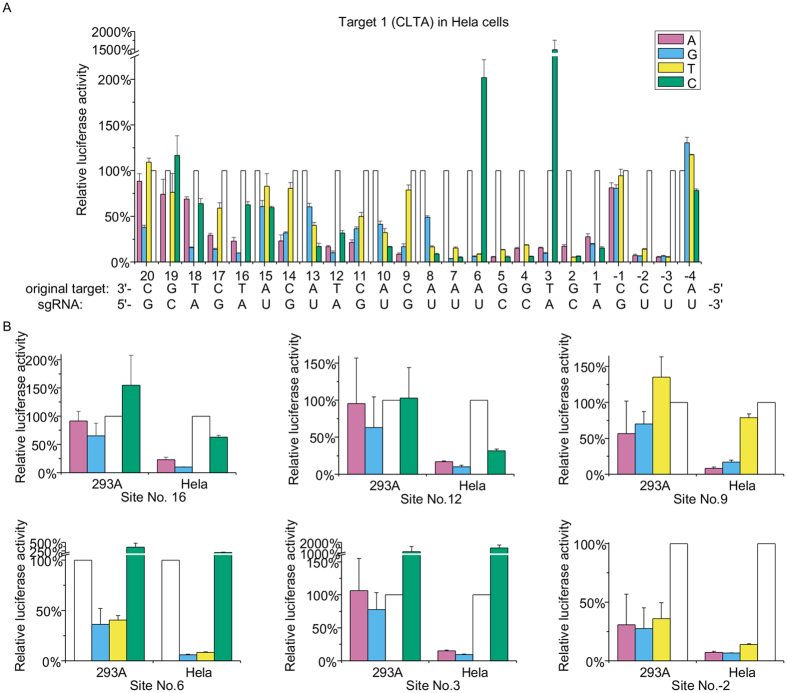
Activity of sgRNA-1 in HEK293 and Hela cells. (**A**) Activity of sgRNA-1 in Hela cells. (**B**) Comparative mismatch discrimination pattern in HEK293 and Hela cells.

In addition to target mismatch, cell type may also play a role in off-target cleavage. To explore this issue, targeting specificity of sgRNA-1 was comparatively examined in HEK293 and Hela cells (Fig. 5). Compared to HEK293 cells, significantly enhanced off-target discrimination was revealed in Hela cells, in both the seed and the non-seed-region. For example at position +16, while off-target cleavage efficacy of rA:dG mismatched target is 65.2% in HEK293 cells; in Hela cells, the cleavage efficacy is only 9.9%. Despite this difference, a similar specificity profile was however revealed for both cells. rA:dC mismatched target is most tolerated, followed by rA:dA mismatched target and rA:dG mismatched target. Among the 24 target positions, similar specificity profiles were observed in 16 of them, suggesting a general targeting specificity profile for a given sgRNA. Interestingly, while the cleavage-enhancing mismatches were also observed at position +3 and +6, NAG PAM was not found active in Hela cells.

**Figure 6:**
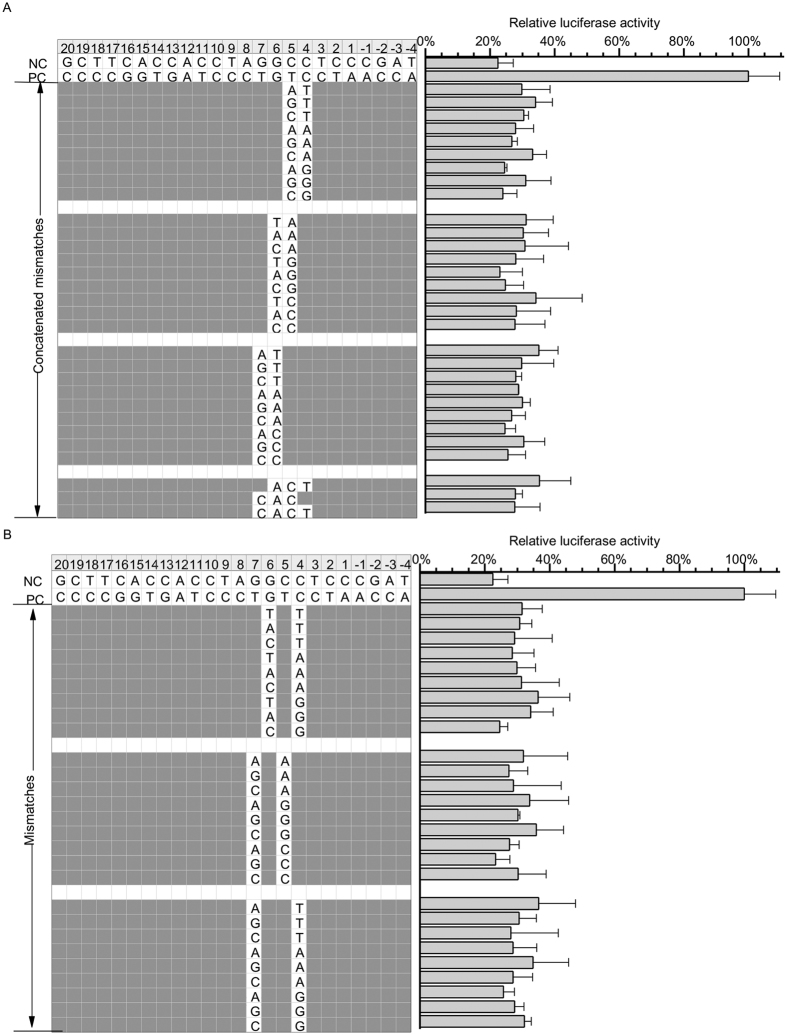
Activity of target sites with multiple mismatches. Relative luciferase activities were measured with 57 mismatched target sites bearing two or more mismatches. Mismatched target nucleotides are indicated.

Having characterized a mismatch-sensitive “core” sequence, we speculated that this property may be used in gene-specific sgRNA design. As some of the mismatches are still tolerated in this region, we hypothesized that it is possible to further decrease or abolish off-target cleavage by introducing multiple mismatches. To this end, two or more mismatches were systematically made in the target sites of sgRNA-2. In total, 57 target plasmids were generated, and tested for potential off-target effects (Fig. 6). The results showed that all the targets bearing two or more mismatches were unable to be cleaved by sgRNA-2. These results indicate that multiple mismatches in the “core” sequence can significantly increase target specificity of a sgRNA and suggest that, it is possible to increase target specificity by choose sgRNA bearing multiple mismatches to potential off-target sites.

Taking together, our data indicate that target specificity of a sgRNA may be determined by a variety of mechanisms, depending on its interactions with target DNA, neighbouring protein domains, and solvent molecules. This is of particularly notable for a “core” sequence within the PAM-proximal region. Being located in a conformation restricted region makes this sequence highly sensitive to target mutation, a property might be exploited in gene-specific sgRNA design. To maximize target specificity of a sgRNA, we suggest that potential ‘off-target’ genomic sites should be examined by considering the following guidelines: (1) potential ‘off-target’ sites should not be followed by a NGG or NAG PAM sequences; (2) global sequence similarity between sgRNA and potential ‘off-target’ sites should be minimized; (3) single or multiple target mismatch in the “core” sequence is preferred.

## Materials and Methods

### Oligonucleotides and plasmids

All DNA oligonucleotides were synthesized by Invitrogen (Beijing, China). pCas9, psgRNA and pTarget plasmid were provided by Biomics Biotechnologies (China, Nantong).

Single-nucleotide mutated target fragment was cloned into linearized pTarget plasmid. The resulting fusion pTarget plasmid was sequenced for verification.

### sgRNA activity assay

Human embryonic kidney (HEK293) cells were grown in Dulbecco’s modified Eagle’s medium (Corning) supplemented with 10% fetal bovine serum (Life Technologies), 100 units/ml penicillin and 100 μg/ml streptomycin (Life Technologies, Gibco). The cells were seeded into 48-well plates at a density of ~2.5 × 10^4^ cells/well one day before transfection. pCas plasmid (200 ng/well), psgRNA plasmid (200 ng/well), and pTarget plasmid (30 ng/well) carrying a target site of tested sgRNA was transfected into HEK293 cells at approximately 70% confluence, together with pRL-TK reference vector (5 ng/well). Activity of both luciferases was determined by a fluorometer (Synergy HT, BioTek, USA) before the *firefly* luciferase activity was normalized to *renilla* luciferase for each well. The cleavage efficacy of sgRNA was calculated by comparison with a sample without sgRNA treatment. All experiments were performed in triplicate and repeated three times.

### T7E1 assay

Human embryonic kidney cells stably expressing Cas9 (HEK293 + Cas9) were grown in Dulbecco’s modified Eagle’s medium (Corning) supplemented with 10% fetal bovine serum (Life Technologies), 100 units/ml penicillin and 100 μg/ml streptomycin (Life Technologies, Gibco). The cells were seeded into 24-well plates at a density of ~4 × 10^4^ cells/well one day before transfection. psgRNA plasmid (500 ng/well) was transfected into HEK293 + Cas9 cells at approximately 50% confluence following the manual of Lipofectamin 2000 (Life Technologies). Cells were collected 72 h after transfection and genomic DNA was extracted (Tiangen TIANamp Genomic DNA Kit, China). T7E1 assay was conducted following the manual of V-Solid T7 endonuclease I Kit (Beijing, China). All experiments were performed in triplicate and repeated three times.

## Additional Information

**How to cite this article**: Zheng, T. *et al*. Profiling single-guide RNA specificity reveals a mismatch sensitive core sequence. *Sci. Rep.* **7**, 40638; doi: 10.1038/srep40638 (2017).

**Publisher's note:** Springer Nature remains neutral with regard to jurisdictional claims in published maps and institutional affiliations.
